# The Brain Retains: Nonhuman Primate Models for Pediatric HIV-1 in the CNS

**DOI:** 10.1007/s11904-020-00503-4

**Published:** 2020-05-10

**Authors:** Veronica Obregon-Perko, Katherine Bricker, Ann Chahroudi

**Affiliations:** 1 Department of Pediatrics, Emory University School of Medicine, Atlanta, GA USA; 2 Yerkes National Primate Research Center, Emory University, Atlanta, GA USA; 3 Emory+Children’s Center for Childhood Infections and Vaccines, Atlanta, GA USA

**Keywords:** Pediatrics, AIDS, Nonhuman primates, CNS, Neurocognitive, Reservoir

## Abstract

**Purpose of Review:**

Perinatal HIV-1 infection is associated with an increased risk for neurologic impairments. With limited access to clinical specimens, animal models could advance our understanding of pediatric central nervous system (CNS) disease and viral persistence. Here, we summarize current findings on HIV-1 CNS infection from nonhuman primate (NHP) models and discuss their implications for improving pediatric clinical outcomes.

**Recent Findings:**

SIV/SHIV can be found in the CNS of infant macaques within 48 h of challenge. Recent studies show an impermeable BBB during SIV infection, suggesting neuroinvasion in post-partum infection is likely not wholly attributed to barrier dysfunction. Histopathological findings reveal dramatic reductions in hippocampal neuronal populations and myelination in infected infant macaques, providing a link for cognitive impairments seen in pediatric cases. Evidence from humans and NHPs support the CNS as a functional latent reservoir, harbored in myeloid cells that may require unique eradication strategies.

**Summary:**

Studies in NHP models are uncovering early events, causes, and therapeutic targets of CNS disease as well as highlighting the importance of age-specific studies that capture the distinct features of pediatric HIV-1 infection.

## Introduction

Globally, 1.7 million children are currently living with HIV-1 infection, with 160,000 new infections occurring annually [1]. The majority of new pediatric infections occur through mother-to-child transmission (MTCT) in utero, intrapartum, or post-partum through breast milk. While the mechanisms are not completely defined, HIV-1-infected infants experience a rapid progression of disease as compared to infected adults, with over 50% of HIV-1-infected children dying before the age of two in the absence of antiretroviral therapy (ART) [2]. ART has greatly reduced HIV-1-related morbidity and mortality but, alone, cannot purge the viral reservoir that is seeded early in infection. In the majority of HIV-1-infected individuals, interruption of ART leads to viral rebound, making daily adherence to medication a lifelong requirement to control virus replication. A handful of pediatric cases of prolonged ART-free remission have been reported, reflecting opportunities of early ART initiation, a strategy that will likely not be applicable to the majority of ongoing infections worldwide [3–5].

Consequences of HIV-1 infection that impact non-AIDS aspects of health are an ongoing challenge in clinical care. A vast range of neurological complications, collectively termed HIV-associated neurocognitive disorders (HANDs), have been reported in HIV-1-infected individuals. Even with ART, HAND is associated with greater risk for disease progression and poorer morbidity [6]. It is estimated that up to 50% of ART-treated children will develop neurologic complications [7–11]. Clinical manifestations include mild to severe neurocognitive impairment, delays in motor development, and behavioral psychiatric conditions such as depression and attention-deficit hyperactivity disorder (ADHD). Progressive encephalopathy, described to compromise brain growth, is a prominent and severe presentation in untreated pediatric infection [12]. In fact, the incidence of encephalopathy is higher in infants than adults in the first year of infection, which may be reflective of pathologic events during fetal and early postnatal brain development [13]. While early diagnosis and ART initiation halts and partially reverses progression, static encephalopathy can persist after treatment [14].

The direct cause of developmental disorders is unclear but may be due to factors such as poor penetrance of ART to the central nervous system (CNS) leading to continuous unchecked viral replication in the brain, chronic neuroinflammation, or neurotoxic effects of long-term ART treatment. While these factors have been explored in adults, there is a critical need to further understand these aspects of disease in children who are exposed to HIV and ART during periods of rapid brain development and are often infected orally through breastfeeding, an understudied yet relevant and dominant mucosal transmission route in pediatric infection [15]. Animal models are an important means to address these questions, with the advantage of overcoming challenges faced when using human samples such as limited access to anatomical sites, small sample volumes, and lack of control over experimental variables (i.e., viral dose, transmission route, and time of ART initiation and duration).

Nonhuman primate (NHP) models of HIV/AIDS have long been a powerful platform that have advanced our understanding of HIV transmission, pathogenesis, and persistence. While investigations of pediatric HIV-1 CNS infection using this animal model are limited, findings from these studies are uncovering key differences from adults in neuropathogenesis that could inform advancements in pediatric care (summarized in Table 1). The purpose of this review is to provide insights recently gained from NHP models of pediatric infection of the CNS as well as discuss their implications for the future development of therapies and cure strategies for children living with HIV-1.

**Table 1:** Key CNS disease findings from pediatric NHP studies

Study	Age at challenge	Macaque species	Infection route	Virus	Dose	Key CNS finding
Amedee et al [32•]	3–8 weeks	*M. mulatta*	Oral	SIV_mac251_	5 × 10^4^ TCID_50_	SIV DNA was detectable in the cerebellum by 48 h post challenge, but SIV RNA is largely undetectable in both brain tissue and CSF.
Hessell et al [30•]	1 month	*M. mulatta*	Oral	SHIV_SF162P3_	50% AID_50_	Viral DNA, but not RNA, was detectable in the cerebellum by 24 h post challenge.
Delery et al [37••]	0–3 months	*M. mulatta*	i.v. and intrarectal	SIV_mac251_, SIV_mac239_, SIV_ΔB670_, or SIV_0302_	Variable	Incidence of encephalitis was age-dependent. Reduced susceptibility to encephalitis in neonates was attributed to a less permeable BBB and a lower frequency of CCR5+ cells in the brain as compared to juvenile/adult macaques.
Lane et al [39]	In utero^a^	*M. mulatta*	Direct fetal inoculation by i.p. injection	SIV_mac251_	10^3^ TCID_50_	Viral DNA, RNA, and protein is widely detectable throughout the fetal brain, but did not localize around vessels by 15 d.p.i. Virus was found most frequently in the cortical white matter. Frequency of virally-infected cells in the brain was lower than juveniles/adults and encephalitis was less common.
Westmoreland et al [38]	1 day	*M. mulatta*	i.v.	SIV_mac251_, SIV_mac239_, or SIV_mac239/316_	20 ng p27/kg	Detected SIV DNA across multiple brain regions but reported lower levels of viral protein or RNA in neonates compared to juveniles/adults. SIV-infected cells were identified near vessels in brain.
Mavigner et al [69••]	4–5 months	*M. mulatta*	Oral	SIV_mac251_	10^5^ TCID_50_	Comparable levels of viral RNA in the brain of ART-suppressed and viremic infants, a finding distinct from adult macaques. Poor ART penetrance to the brain and low to undetectable levels of ART drugs in the CSF, as measured by LS-MS/MS.
Curtis et al [51]	1 week	*M. mulatta*	i.v.	SIV_mac251_	100 TCID_50_	Neuronal reduction of the hippocampus in SIV-infected infants compared to healthy controls that was more pronounced in orally-infected vs. i.v.-infected infants.
Carryl et al [52••]	9–20 weeks	*M. mulatta*	Oral	SIV_mac251_	5000 TCID_50_	
Kinman et al [63]	36 days	*M. nemestrina*	i.v. or intrathecal	HIV-2_287_	10^3^ TCID_50_	Viral RNA was detectable in CSF at multiple timepoints post infection. Neurocognitive development was delayed in all macaques, but was more severe in i.v.-infected infants.
Worlein et al [64]	In utero^b^	*M. nemestrina*	i.v. inoculation of dam	HIV-2_287_	10, 10^3^, or 10^4^ TCID_50_	Delayed motor and cognitive development in infected infants compared to healthy age-matched controls.

^a^Infections were timed at GD 65 (early 2nd trimester), 110 (early third trimester), or 130 (mid third trimester)

^b^Pregnant dams were i.v. inoculated during the third trimester of pregnancy

## SIVs and SHIVs in HIV Research

Asian NHPs, namely rhesus (*Macaca mulatta*), pigtailed (*Macaca nemestrina*), and cynomolgus (*Macaca fascicaluris*) macaques, have become the most commonly used and widely accepted animal models for HIV-1 infection [16–18]. Additionally, neurodevelopment is similar between infant humans and macaques, making them suitable for studies of neurologic disease [19–21]. With some variability between species, most Asian macaques are readily infected with simian immunodeficiency virus (SIV) and model key viral and immune features of infection such as gradual CD4+ T cell depletion, progression to AIDS, suppression of viremia with ART, and effective transmission through mucosal routes [22]. The low prevalence of CNS disease with the most commonly used strains of SIV can present a challenge for neuropathogenesis studies in macaques, however. As such, SIV and HIV neurotropic strains have been developed and optimized in macaque species to yield more consistent outcomes of CNS disease (Table 2; further described in “NHP Models of Accelerated CNS Disease”).

**Table 2:** Selected viruses used to study HIV in the CNS

SIV_mac251_ [99]. Highly pathogenic uncloned isolate (viral swarm) that readily infects lymphocytes and macrophages; demonstrates invasion of the CNS, but does not consistently cause CNS disease.	
SIV_mac239_ [99]. Prototypical pathogenic molecular clone, readily infects lymphocytes but poorly infects monocytes/macrophages; infects the CNS.	
SIV_mac239/316_ [38]. Derivative of SIV_mac239,_ differs from SIV_mac239_ by 8 AA resulting in more productive infection in monocytes/macrophages.	
HIV-2_287_ [63, 100]. Highly pathogenic viral strain developed from culture supernatant of HIV-2_EHO._ Infected infant pigtailed macaques show accelerated CNS disease, including high CSF viral loads and neurocognitive impairments.	
SHIV_SF162P3_ [101]. Strain of simian human immunodeficiency virus (SHIV) that results in high acute phase viremia and can lead to simian AIDS in rhesus macaques. Chimeric virus allows direct testing of therapies targeting the HIV envelope.	
SIV/17E-Fr + SIV/ΔB670 [102]. Dual tropic virus swarm that results in full immunosuppressive disease and encephalitis in pigtailed macaques. *SIV/17E-*Fr alone is a neurovirulent molecular clone composed of SIV_mac239_ backbone containing Env, Nef, and 3′ LTR genes of viral isolate obtained from a macaque with fulminant encephalitis (SIV/17E-Br). *SIV/ΔB670* was developed through co-culture of lymph node tissue from an SIV-infected monkey (B670) with stimulated primary human mononuclear cells and is immunosuppressive.	

Although SIV-macaque models have been widely used for studies of HIV-1 transmission, immunopathogenesis, vaccination, and cure, differences in SIV and HIV-1 can make it difficult to address certain experimental questions. For instance, the efficacy of vaccines or entry inhibitors developed against the HIV-1 envelope, a site of heavy divergence between SIV and HIV-1, cannot be directly tested using an SIV challenge. Simian/human immunodeficiency viruses (SHIVs) expressing HIV-1 Env glycoproteins or proteins targeted by antiretrovirals have been constructed to address this gap in translational studies. While initial chimeric variants showed poor replication in macaques, the pathogenicity of next-generation SHIVs has been improved by serial-passage and enhanced affinity for macaque entry receptors [23, 24•, 25, 26]. Studies demonstrating neuroinvasion of SHIV variants in the pediatric NHP setting are limited. The value of these viruses in pre-clinical studies warrants their further characterization and development for investigations of CNS infection.

It is important to consider that studies to date, and described in this review, encompass pediatric NHP models using a range of species, age at challenge, route of infection, virus, and dose (Tables 1 and 2). Thus, it is key to balance reported findings with the suitability of the model used to address the aspect of CNS infection under investigation (i.e., neuroinvasion, target cells of infection, reservoirs, neurological symptoms, etc.).

## CNS Entry and Localization

### Timing

Studies in adult macaques have yielded conflicting models of timing for systemic dissemination after mucosal challenge. While studies of SIV vaginal transmission have reported viral production confined at the port of entry for days before spread, others using the same model have detected virus in draining lymph nodes within 24 h [27–29]. Evidence from orally infected infant rhesus macaques shows rapid dissemination of virus to proximal tissues, with viral RNA found in the periphery by 2 days post-challenge [30•, 31]. Thus, after infection across the oral mucosa, the virus quickly gains access to draining lymph nodes of the head and neck which could mediate early invasion of other anatomical sites [30•, 31].

While previous reports have shown SIV in neonatal macaque brains within 3 to 7 days of in utero or i.v. challenge, recent studies of oral transmission have demonstrated even earlier infiltration of this site. In a study of 15 infant macaques infected with SIV_mac251_ between 3 to 8 weeks of age, SIV DNA was detectable in the brain as early as 48 h after challenge [32•]. 40% of infants analyzed at 48 h had detectable SIV DNA in the cerebrum, with the percentage rising to 67% by 72 h post-challenge. Similar kinetics of viral DNA distribution in infant macaques have also been reported following challenge with SHIV_SF162P3_, with viral DNA detectable in the cerebellum within 1 day of challenge [30•]. Both studies also measured RNA levels to assess if DNA-positive tissues were sites of productive infection. In the SIV_mac251_-infected macaques, viral RNA was only detected in 1/15 macaque brains and no RNA was detected in the CSF within 96 h. SHIV_SF162P3_ RNA was only found in the cerebellum at 14 days but not at 1 day post-challenge. Undetectable levels of RNA in the CNS of these animals before 72 h post-infection may be reflective of recent immigration of infected cells to this site, before localized replication and spread.

Understanding how and when the virus disseminates into the CNS in pediatric HIV-1 infection could reveal how long the window of opportunity is to impede neuroinvasion. The use of oral infection in these described infant macaque studies makes the findings of particular relevance for breastfeeding transmission, the route by which the majority of new pediatric HIV-1 infections are now acquired.

### Mechanisms of Entry

The most widely accepted and supported mechanism of CNS entry by HIV/SIV is thought to be chemokine-mediated migration of virally-infected lymphocytes and monocytes across the blood-brain barrier (BBB), where they can release virus to resident target cells [33, 34]. Although not as well-characterized, other mechanisms have been proposed, including entry of cell-free virus through a disrupted BBB or direct infection of cells that line the BBB [35].

Studies of viral CNS entry processes in perinatal infection are limited but suggest that invasion events may differ by developmental stage. In infants and neonates, developing cerebral vessels are more susceptible to damage from drugs, toxins, or neuroinflammation, which could lead to barrier dysfunction [36]. Such damage could provide an opportunity for CNS invasion by free virus. Delery et al. recently demonstrated that the BBB of neonatal rhesus macaques actually remains fairly impermeable during SIV infection [37••]. This would support the role for entry mediated by a “Trojan Horse” or infection of BBB-lining cells. In line with this hypothesis is the previous identification of SIV-infected cells localized to blood vessels in the brains of neonatal macaques infected intravenously [38]. Interestingly, virus was rarely found in these areas in fetal macaques infected in utero [39]. Taken together, these studies provide preliminary evidence for the notion that invasion events could differ by developmental stage at time of infection or transmission route. It is worth noting a limitation of each of these studies was the use of a single technique to draw conclusions on mechanisms of viral invasion. There is more to be learned from carefully designed studies that utilize a combination of ISH, permeability markers, and cell tracking to delineate the major mechanism(s) of viral entry into the neonatal brain with the long-term goal of identifying targets for pre- or post-exposure prophylaxis.

### Sites of Infection

In infant macaques, the prevalence of CNS infection is similar to that of juveniles and adults, but differences in the distribution of virally infected cells have been reported. In fetal rhesus macaques infected in utero with SIV_mac251_, virus-positive cells—identified by DNA, RNA, and protein—were present within the meninges, basal ganglia, stroma of choroid plexus, external granular layer of the cerebellum, cortical plate, and cortical white matter within 15 days post-infection [39]. Of these locations, virus was most frequently found in the cortical white matter. While SIV can be found across this region in juvenile and adult macaques, this is typically only under conditions of encephalitis [40, 41], that was not seen in the infants. An additional study, in which newborn macaques were infected i.v. with SIV_mac251_, SIV_mac239_, or SIV_mac239/316_, also found detectable SIV DNA across multiple overlapping brain regions [38]. Here, infected cells were frequently identified in the cortical gray matter, an area less dominated by SIV infection in older animals [40, 42].

Altered viral distribution in fetal, neonatal, and juvenile infection may reflect expansion in cell tropism at early developmental stages. During gestation, glial and neuronal cells are mitotically-active, which contrasts the more static nature of the adult CNS [43]. Brain regions of ongoing cell proliferation in the fetus, which would be rarer in healthy adults, could then become a unique site of viral replication. Yet, the question remains of how such cell types, like astrocytes, could be targets of infection if they have little to no expression of required entry receptors. A recent report showed a paucity of CCR5+ cells within the brain of uninfected neonatal macaques, despite SIV-infected neonates having similar viral DNA and RNA levels in the brain compared to adults [37••]. This suggests the possibility of alternative means for viral spread in a setting of limited CCR5 availability, such as through the formation of virologic synapses, which could favor infection of cells that would otherwise be spared from direct receptor-mediated infection [44]. Whether astrocytes can support productive infection or reservoir establishment in vivo is still debated, but the presence of virus or viral products in these and other cells, such as microglia and perivascular macrophages, could contribute to bursts of viral release or inflammation from persistent antigen exposure [45–48]. Extending studies to identify anatomical foci and target cells of HIV/SIV perinatal infection could guide targeted delivery of therapeutics into these regions of early viral replication.

## Neuropathogenesis

### Histopathological Findings

Differences in virus localization throughout the brain in pediatric infection, as described in the previous section, raises the possibility of altered or accelerated pathogenesis of neurologic disease induced by HIV/SIV in this age group. Previous histological findings in rhesus macaques infected with SIV in utero or shortly after birth show brain pathologies that closely resemble those seen in HIV-1-infected children [49, 50]. Decreased brain growth, evident after 2 months of infection, has been reported in SIV-infected neonatal macaques [38]. Perivascular infiltrates of mononuclear cells, mineralization of vessels in the basal ganglia, and proliferation of glial cells could also be seen within 3 weeks of infection [38, 39]. Although such pathologies were generally associated with regions where virus was detected, it is unclear whether lesions or delayed brain growth are the result of direct or indirect effects of the infection.

A growing body of work is providing anatomical evidence for neurological impairments and disease observed in pediatric HIV-1 infection. Newborn rhesus macaques infected i.v. with SIV_mac251_ have presented with dramatic reductions in immature neurons and the pyramidal neuron population within the hippocampus at 3 months post-infection [51]. A follow-up study by Carryl et al. reported more pronounced pathological findings when animals were infected orally, although orally infected animals were also older [52••]. Reductions in hippocampal myelination were also evident [53]. Loss of hippocampal neuronal cell types and demyelination could explain the mechanisms underlying the rapid neurocognitive and neuromotor decline sometimes seen in pediatric HIV-1 patients, including deficits in memory and the onset of multiple sclerosis-like illness [54, 55]. Congruency with clinical findings further validates the use of NHP models to evaluate and improve the course of HIV-1 CNS infection in children.

### Encephalitis

Despite the presence of virus in the CNS and the incidence of neurologic complications in pediatric HIV-1 infection, reports of encephalitis are scarce [56–58]. SIV-infected infant macaques also rarely present with multi-nucleated giant cells in the brain, one histological hallmark of encephalitis [39, 59]. These findings are particularly surprising when one considers the context of perinatal infection, characterized by high plasma viral loads and rapid disease progression. Seeking to address this paradox, a recent retrospective analysis of over 100 SIV-infected rhesus macaques uncovered that incidence of encephalitis is age-dependent [37••]. In this study, no signs of encephalitis were seen in any of the animals infected as neonates (*n* = 51), with the earliest case observed in an animal infected at 4 months of age. Incidence in juveniles and adults, however, reached approximately 25%. While more direct investigations are needed to uncover features that influence encephalitis susceptibility, these findings highlight the importance of age-spectrum studies which could uncover not only mechanisms underlying accelerated disease progression but also features of protection from HIV-associated pathologies.

### NHP Models of Accelerated CNS Disease

Previous studies have shown that about 20–36% of SIV_mac_-infected rhesus macaques exhibit neuropathological lesions and symptoms of CNS disease, a frequency similar to HIV-1-infected patients [60]. Thus, while SIV_mac_ infection of rhesus macaques provides a strong homolog to HIV-1 infection of humans, the infrequency of neuropathology makes it challenging to use this system for deep investigations of CNS infection, such as uncovering the cause(s) of neuronal dysfunction or loss and their impact on behavior and cognitive abilities. Animal models of accelerated and consistent CNS disease could allow studies of shorter duration with fewer animals to interrogate these processes.

Zink et al. developed such a system by co-inoculating pigtailed macaques with two SIV strains: neurovirulent SIV/17E-Fr and immunosuppressive SIV/DeltaB670 [61]. Over 90% of infected animals developed CSF viral loads on the order of 10^6^ copies/ml by 10 days post-inoculation. Within 3 months, animals progressed to AIDS, developed encephalitis, and displayed neuronal damage. Importantly, a significantly lower prevalence of encephalitis was seen in rhesus or cynomolgus macaques given the same co-inoculation, suggesting that host genetic factors also play a role in neurological disease outcome in this model [61, 62]. It could be of great value to apply this system to fetal or neonatal pigtailed macaques to assess disease events and their impact on neurodevelopment, as has been done previously with HIV-2_287_ [63, 64]. In addition to high viral loads and neurological lesions, animals in these studies infected in utero or at 1 month of age showed delays in motor and cognitive development. While these NHP models of rapid CNS disease progression may not wholly reflect immune and viral events seen in the slower progression of HIV-1 infection, such models could still deepen our understanding of the sequence of neuropathologic events as well as provide a platform for testing drug candidates that can improve or preserve neurologic functions in pediatric HIV-1 infection.

## The CNS as a Latent Reservoir

### Viral Persistence on ART

The BBB exists to tightly regulate the entry of solutes and inhibit invasion of pathogens into the CNS; however, it is this feature that contributes to low ART drug penetrance as well as limited immunosurveillance in the CNS [65, 66]. Such circumstances could provide a sanctuary for virally-infected cells and permit ongoing replication. Indeed, untreated and ART-suppressed macaques have comparable frequencies of cells harboring SIV_mac251_ RNA or DNA in brain tissue [24•, 67, 68]. Recently, our laboratory reported similar findings comparing viremic and ART-suppressed orally-infected infant rhesus macaques [69••]. In addition, we also observed low to undetectable ART drug levels across the brain in all animals, including the cortices, frontal lobe, and basal ganglia. While these observations clearly demonstrate poor clearance of SIV in the brain, whether the CNS could serve as a functional latent reservoir has long been a source of controversy.

### Findings from HIV-1-Infected Patients

A growing body of evidence supports the CNS as an anatomical reservoir in HIV-1 infection. HIV-1 RNA has been detected in the CSF but not in the blood of patients on ART [70–74]. This discordance in CSF and plasma viral loads, termed CSF viral escape, raises the possibility of ongoing low-level replication or intermittent bursts of virus production in the CNS even in the absence of systemic HIV replication [75]. CSF viral escape is more prevalent in adults with neurologic symptoms or poorer neurocognitive performance, as is higher levels of persistent HIV DNA in the CSF of adults with viremia suppressed by ART [70, 72, 76, 77•]. Deep-sequencing analysis has revealed compartmentalized viral evolution within the CSF, evidenced by viral populations genetically distinct from those in the blood and capable of contributing as an independent source of viral rebound within the CSF after ART interruption [78–82]. Extensive analyses in this area are generally lacking for perinatal infection. One study has documented CSF compartmentalization by 3 years of age in up to 50% of ART-naïve children infected with HIV-1 subtype C [83]. Here, independent replication in the CNS was proposed to occur by early sequestration of a single transmitted variant to the CNS or by emergence of CNS-adapted variants in later stages of infection.

### Macrophages and Microglia as Viral Reservoirs

Resting memory CD4+ T cells are thought to be the predominant source of replication-competent reservoirs in blood and peripheral tissues. However, the genome of rebounding virus cannot always be phylogenetically traced back to proviral genomes in resting CD4+ T cells, indicating the possible existence of a non-CD4+ T cell pool of persistent virus [84–86]. Viral DNA and RNA have been found in brain macrophages and resident microglia of SIV-infected infant and adult rhesus macaques as well as in HIV-infected patients [31, 38, 40, 45, 69••, 87–89]. Adapting the quantitative viral outgrowth assay (QVOA) to brain macrophages, Avalos et al. showed these cell types harbor replication-competent virus in ART-suppressed pigtailed macaques [90••]. In this same NHP model, treatment with latency reversing agents in vivo led to focal reactivation of viral reservoirs in brain macrophages that, in some animals, occurred independently from the periphery [91]. Experimental CD4 depletion in SIV-infected rhesus macaques has been shown to result in productive infection of macrophages and microglia, with peripheral set point viral loads reaching levels two logs higher than undepleted controls [88, 92]. Taken together, these studies demonstrate myeloid cells in the brain can be targets of SIV infection and harbor replication-competent virus even in the setting of long-term ART treatment.

Advancements in ART delivery to the CNS will likely be insufficient for eradication in myeloid cell types, as many ART drugs already used in the clinic show limited efficacy in microglia and macrophages [93]. The myeloid lineage also presents a particular challenge for cure strategies as they can be long-lived, are capable of self-renewal, and are not efficiently killed by CD8+ T cells [94–96]. Thus, efforts for viral clearance, like shock and kill strategies, should also be evaluated for activity against myeloid cells and confirmed in infant models of HIV-1 infection.

## Conclusions

**Fig. 1:**
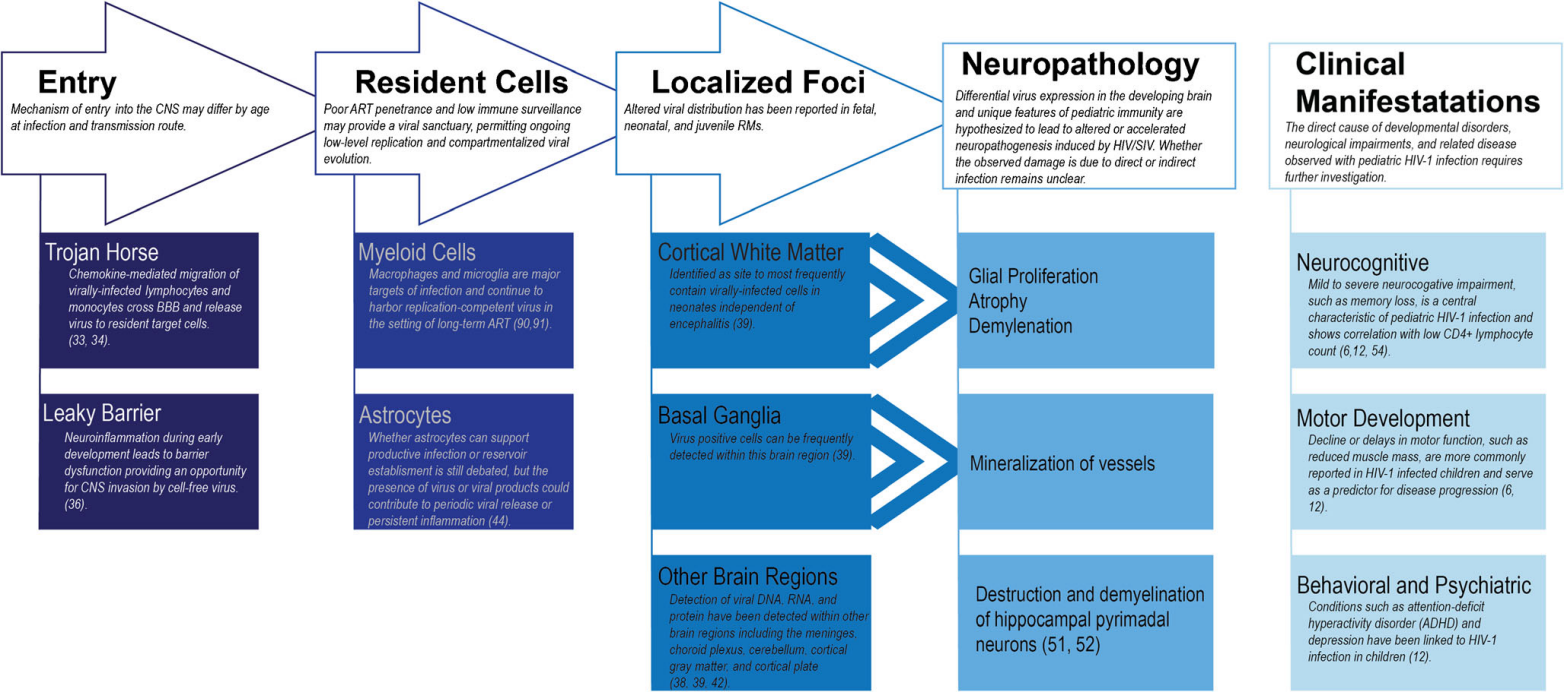
Schematic of proposed interaction between HIV/SIV and the CNS

NHP models have provided valuable insights into HIV-1 CNS infection, including timing of neuroinvasion, anatomical links to specific neurologic impairments, and identification of cell types harboring latent virus (see Table 1 and Fig. 1). However, there is much to be learned in these areas for perinatally-infected children. While findings in adult humans and NHPs can pave the way for progress in the treatment of CNS disease, it is still critical these studies be validated in pediatric models. Immune and virologic features unique to pediatric infection could impact mechanisms that promote persistence or disease [97•]. For instance, our lab has shown naive CD4+ T cells are the major contributor to the total CD4+ T cell reservoir in SIV-infected infant rhesus macaques, in contrast to central memory CD4+ T cells in adult macaques [69••]. In addition, infant rhesus macaques have a higher baseline turnover rate of monocytes, which further increases during SIV infection and is associated with rapid progression to AIDS [98]. Such findings highlight the necessity for pediatric-focused studies to ensure cure strategies and treatments for neurological impairments will be relevant in this age group.
